# Rapid host switching in generalist *Campylobacter* strains erodes the signal for tracing human infections

**DOI:** 10.1038/ismej.2015.149

**Published:** 2015-08-25

**Authors:** Bethany L Dearlove, Alison J Cody, Ben Pascoe, Guillaume Méric, Daniel J Wilson, Samuel K Sheppard

**Affiliations:** 1 Nuffield Department of Medicine, Experimental Medicine Division, University of Oxford, Oxford, UK; 2 Department of Zoology, University of Oxford, Oxford, UK; 3 College of Medicine, Institute of Life Science, Swansea University, Swansea, UK; 4 MRC CLIMB Consortium, Institute of Life Science, Swansea University, Swansea, UK; 5 Wellcome Trust Centre for Human Genetics, University of Oxford, Oxford, UK

## Abstract

*Campylobacter jejuni* and *Campylobacter coli* are the biggest causes of bacterial gastroenteritis in the developed world, with human infections typically arising from zoonotic transmission associated with infected meat. Because *Campylobacter* is not thought to survive well outside the gut, host-associated populations are genetically isolated to varying degrees. Therefore, the likely origin of most strains can be determined by host-associated variation in the genome. This is instructive for characterizing the source of human infection. However, some common strains, notably isolates belonging to the ST-21, ST-45 and ST-828 clonal complexes, appear to have broad host ranges, hindering source attribution. Here whole-genome sequencing has the potential to reveal fine-scale genetic structure associated with host specificity. We found that rates of zoonotic transmission among animal host species in these clonal complexes were so high that the signal of host association is all but obliterated, estimating one zoonotic transmission event every 1.6, 1.8 and 12 years in the ST-21, ST-45 and ST828 complexes, respectively. We attributed 89% of clinical cases to a chicken source, 10% to cattle and 1% to pig. Our results reveal that common strains of *C. jejuni* and *C. coli* infectious to humans are adapted to a generalist lifestyle, permitting rapid transmission between different hosts. Furthermore, they show that the weak signal of host association within these complexes presents a challenge for pinpointing the source of clinical infections, underlining the view that whole-genome sequencing, powerful though it is, cannot substitute for intensive sampling of suspected transmission reservoirs.

## Introduction

*Campylobacter jejuni* and *Campylobacter coli* are zoonotic pathogens with broad host ranges, carried, apparently asymptomatically, as part of the gut microbiota of a range of wild and domesticated mammal and bird species. Common carriage in the gut of animals and poultry farmed for meat leads to numerous opportunities for contamination of food products, which may take place at various points from farm to fork (Neimann *et al.*, 2003). Human-to-human transmission is rare (Allos, 2001), hence *Campylobacter* infection in humans tends to be sporadic, and seldom manifests as outbreaks except when a single point source results in direct transmission to many people, for example, via contaminated drinking water (Palmer *et al.*, 1983; Pebody *et al.*, 1997; Engberg *et al.*, 1998; Clark *et al.*, 2003).

The sources of human *Campylobacter* infection have been well characterized at the population level. Genetic analysis, particularly using seven-locus multilocus sequence typing (MLST) data, has helped to attribute the sources of clinical infections by exploiting differences in the frequency of *Campylobacter* strains that live in different animal and environmental reservoirs (McCarthy *et al.*, 2007). For example, isolates belonging to related sequence types (STs) from the ST-257 and ST-61 clonal complexes are strongly associated with chicken and ruminants, respectively (Sheppard *et al.*, 2011). Wild bird species also serve as different bacterial niches, generally being colonized by phylogenetically divergent *Campylobacter* lineages regardless of shared geography (Griekspoor *et al.*, 2013). Independent MLST-based studies in England, Scotland and New Zealand found that >95% of human infections are attributable to animals farmed for meat and poultry, with 56–76% of cases attributable to poultry in particular (Wilson *et al.*, 2008; Mullner *et al.*, 2009; Sheppard *et al.*, 2009).

However, at the individual isolate level, there is often considerable uncertainty in source attribution because some of the most common disease-causing strains, notably those belonging to the ST-21, ST-45 and ST-828 clonal complexes, are regularly isolated from multiple animal species. This means that quantitative attribution to a single host reservoir is difficult using MLST data alone. The reason for the apparently broader host range of these lineages is not currently understood and may reflect true host generalism (Gripp *et al.*, 2011; Sheppard *et al.*, 2014) or the existence of host-restricted sublineages within these clonal complexes.

The increasing availability of whole-genome sequence data provides a potential solution to the challenge of attributing the origin of generalist strains in clinical samples, as a host-specific signal might be gleaned from the remaining 99.8% of the genome outside the seven loci sequenced by standard MLST. Genomics has been used to investigate direct transmission events in a range of bacterial species, including *Staphylococcus aureus* (Harris *et al.*, 2010; Price *et al.*, 2014), *Clostridium difficile* (Eyre *et al.*, 2013) and *Mycobacterium tuberculosis* (Walker *et al.*, 2013). This raises the prospect of enhancing understanding of *Campylobacter* epidemiology, for example, by identifying individual retailers or producers of *Campylobacter*-contaminated food in cases of human infection, and detecting cryptic point source outbreaks (Cody *et al.*, 2013). Ultimately this information can help to inform targeted interventions and reduce the incidence of human campylobacteriosis (Sears *et al.*, 2011).

In this study, we investigated the utility of whole-genome sequence data for improving the accuracy with which human cases can be attributed to particular animal reservoirs by focusing on common strains that are difficult to attribute with MLST. Specifically, we sought to detect fine-scale host-specific genetic structuring within *C. jejuni* ST-21 and ST-45 complex and *C. coli* ST-828 complex isolates sampled from different animal species and to exploit that signal to improve human source attribution. We observed extraordinarily rapid rates of zoonotic transfer within these strains leading to little or no phylogenetic association with host species, limiting the ability of whole-genome sequencing to improve source attribution, but revealing new insight into the transmission dynamics within these common *Campylobacter* strains.

## Materials and methods

### Sequences

The ST-21 and ST-45 (*C. jejuni*) and ST-828 (*C. coli*) clonal complexes were chosen as the focus of the study, because they are among the most common lineages causing human disease but are difficult to attribute to source populations using seven-locus MLST data (Wilson *et al.*, 2008; Sheppard *et al.*, 2009). Isolates from these three clonal complexes were chosen from MLST collections (www.pubmlst.org/campylobacter, Jolley and Maiden (2010)), sequenced and assembled according to the protocols described in Sheppard *et al.* (2013a, 2013b) and Cody *et al.* (2013). In total, 348, 87 and 158 sequences were obtained for the ST-21, ST-45 and ST-828 complexes, respectively (Lefébure *et al.*, 2010; Cody *et al.*, 2013; Sheppard *et al.*, 2013a, b, 2014; see Supplementary Table S1). Isolates belonging to these complexes are predominantly associated with chicken and cattle, from where the majority of animal samples were obtained (13 chicken isolates from each of the clonal complexes, and 7, 9 and 10 cattle isolates for the ST-21, ST-45 and ST-828 clonal complexes, respectively). In addition, three ST-45 complex isolates were included from wild bird species and six ST-828 complex isolates from pigs. Clinical disease isolates (328, 68 and 128 for the ST-21, ST-45 and ST-828 clonal complexes, respectively) were obtained from a surveillance study of samples submitted to the microbiology laboratory of the John Radcliffe Hospital, Oxfordshire, UK.

After Illumina sequencing (Illumina, San Diego, CA, USA), the high coverage short reads were *de novo* assembled using Velvet (Zerbino and Birney, 2008), and the resulting contiguous sequences (‘contigs') stored using BIGSdb (Jolley and Maiden, 2010). These contigs were then compared with the NCTC11168 reference sequence (Genbank accession number: AL111168) to identify genes using a BLAST search (Parkhill *et al.*, 2000; Gundogdu *et al.*, 2007). Only coding genes found in the NCTC11168 reference were used, as intergenic regions can be difficult to align and the coding region still represents 94.3% of the genome (Parkhill *et al.*, 2000). Orthologous genes were defined as homologous genes that had ⩾70% nucleotide identity and <50% difference in alignment length. Genes for all isolates were then aligned using MUSCLE (Edgar, 2004) and concatenated into a single sequence per sample (available from Dryad Digital Repository: http://dx.doi.org/10.5061/dryad.4428s).

### Phylogenetic analysis

The phylogenetic reconstruction software BEAST (Bayesian Evolutionary Analysis by Sampling Trees) v1.7.5 (Drummond *et al.*, 2012) was chosen for implementing the analysis. BEAST performs the Bayesian analysis of aligned sequence data and reconstruction of phylogenies under a variety of coalescent models. The advantage of this Bayesian setting is that by sampling a wide range and number of trees, there is no need to condition on a single tree when making inference, taking into account all of the uncertainty in the topology, branches and evolutionary model. There are a number of Bayesian methods for phylogenetic inference, and here BEAST has two advantages over other similar programs: the relaxed clock substitution model, which allows rates to vary between lineages, and a method for phylogeography (or discrete traits mapping) developed by Lemey *et al.* (2009). This latter approach allows us to treat source populations as if they are distinct geographic entities, and by overlaying isolate source information on the phylogeny, we are able to describe the ancestry of different lineages in the context of host switching.

A constant population size was assumed, and all parameters were scaled in terms of the effective population size, *N*_e_, by fixing it to 1.0. The HKY85 model of nucleotide substitution (Hasegawa *et al.*, 1985) was used with an uncorrelated log-normal relaxed clock and gamma rate heterogeneity with four categories (Drummond *et al.*, 2006). A log-normal prior with a mean of 1.0 and s.d. of 1.25 on the logarithmic-scale was assumed for the transition:tranversion ratio *κ*, and an exponential prior with a mean of 1.0 was utilized for the gamma shape parameter *α*. For the log-normal relaxed clock parameters, a uniform prior between 0.0 and 10.0 was assumed for the mean, and an exponential with mean 1.0 for the s.d. A uniform (Dirichlet) prior was used for the nucleotide frequencies.

We used the phylogeographic model by Lemey *et al.* (2009) to reconstruct zoonotic transmission and infer the source of the infection (host species) along the branches. This allows us to report the posterior probability of any node in the tree being in a particular state (that is, the most likely host of the ancestral lineage) and also to obtain relative rates of transition between states (that is, switching between host species). Given that human-to-human transmission is rare (Allos, 2001), an ambiguity code was set up in the zoonotic model to allow the human isolates to have an ‘unknown' source population. For each human isolate, the other host species in the analysis were given equal prior probability and thus most likely source of the human isolates could be inferred. A uniform prior from 0.0 to 100.0 was assumed for the host migration rate, a gamma prior with mean 1.0 and scale 1.0 for the relative rates of migration and a uniform (Dirichlet) prior for the host population equilibrium frequencies. To calculate the number of discrete zoonotic transmissions across the branches, Markov jumping was performed using BEAST (Minin and Suchard, 2008a; Talbi *et al.*, 2010).

The Markov Chain Monte Carlo was run for 500 million iterations, with samples taken every 10 000 iterations. For each ST, the analysis was repeated twice (three times for ST-21) with different initial values to check convergence and mixing, and these runs were combined for the final results. Unless otherwise stated, the posterior median was used for point estimates and the (2.5%, 97.5%) quantiles for credible intervals. The inferred ancestral host type for each branch in the phylogeny was taken to be the one with the highest posterior probability. Sample dates were not available for all isolates, so the analysis is given in coalescent time (denoted τ) and calibrated to years using an independent point estimate of the mutation rate in *Campylobacter* of 3.23 × 10^−5^ substitutions per site per year from Wilson *et al.* (2009). This estimate was calculated from MLST data but currently represents the best available rate.

### Accounting for ancestral recombination

There is good evidence that novel diversity in *Campylobacter* is generated frequently by the continued movement of genes between lineages, more so even than by the evolution of new variants through mutation (Wilson *et al.*, 2009; Sheppard *et al.*, 2010). High levels of recombination lead to mosaic genomes with differing ancestral histories, and the full evolution of samples cannot be represented by a coalescent genealogy. There are software available (for example, ClonalFrame; Didelot and Falush, 2007) that allow ancestral relationships to be inferred alongside recombination events. However, such methods tend to be limited in their model choice and are often computationally slow, especially for large numbers of genomes.

During preliminary analyses using BEAST (Drummond *et al.*, 2012), a relaxed clock model and gamma site heterogeneity were used to try and account for the effect of recombination. Informally, this assumes that a recombination event with high sequence diversity on a branch is equivalent to that branch having a relatively high mutation rate when compared with branches with no or low diversity recombination events and is a step towards the model underlying ClonalFrame (Didelot and Falush, 2007) with the model flexibility of BEAST (ClonalFrame could not be used to account for recombination because it does not implement the phylogeography model). However, these preliminary analyses diagnosed difficulties in the mixing of the Markov Chain Monte Carlo algorithm underlying BEAST, with multiple runs of the same analysis frequently converging to different topologies.

Despite the fact that *Campylobacter* species are relatively highly recombining, the clonal frame (the genealogy of sites not subject to recombination) can be recovered from whole-genome sequences with high accuracy by maximum likelihood (Hedge and Wilson, 2014). Therefore, we identified and removed the homoplasious sites incompatible with the maximum likelihood tree (Pupko *et al.*, 2000; Guindon *et al.*, 2010). This resulted in a large proportion of variable sites being removed (Table 1). By removing homoplasious sites and retaining only those sites compatible with the clonal frame, we essentially fix the tree topology to that of the clonal frame. This can shorten the deeper branches on the tree. However, the branch lengths are of less direct interest as the estimates of time of switching events is of secondary importance, and we do not expect homoplasy removal to strongly influence estimation of relative rates of transmission between host species—the principal aim of this study.

For the final data set, only the biallelic sites compatible with the inferred phylogeny were included in the alignment, alongside all of the non-variable sites. The removal of homoplasies in this way resolved the mixing issues with BEAST. In addition, to improve computation time for later analyses (as BEAST imputes missing data at each iteration), missing alleles were imputed using ClonalFrameML (Pupko *et al.*, 2000; Didelot and Wilson, 2015).

## Results

### Fine-scale phylogenetic structure within STs

**Figure 1:**
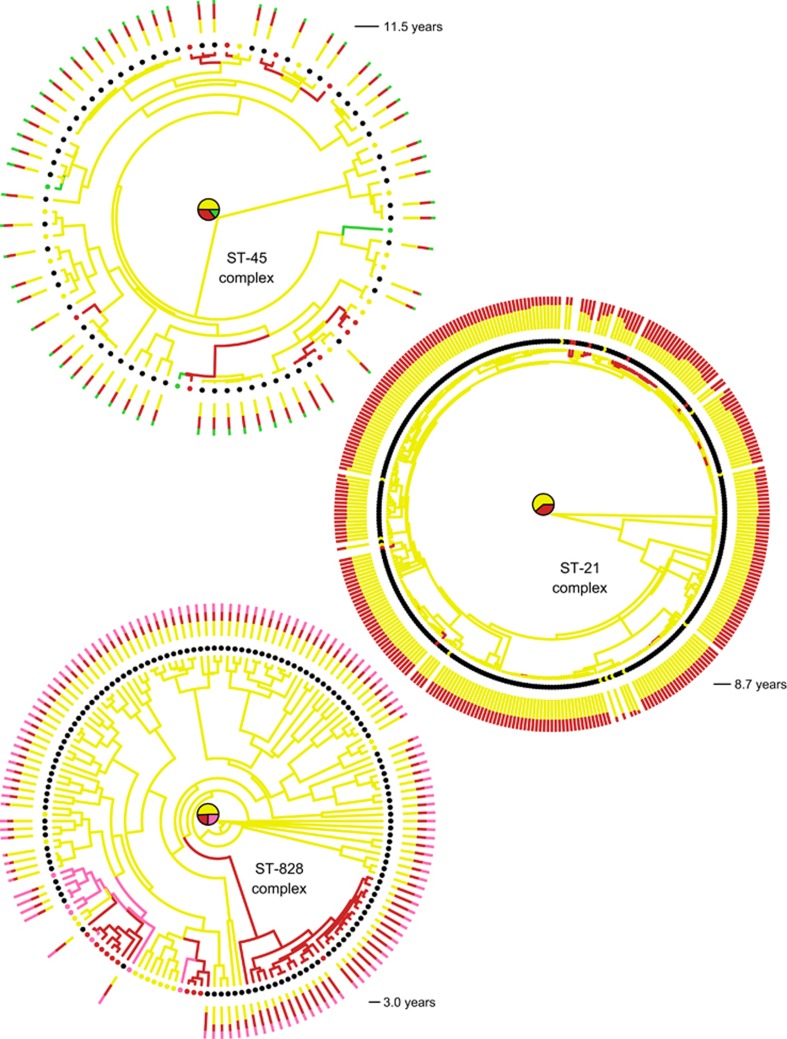
Maximum clade credibility trees for the ST-21, ST-45 and ST-828 complexes. Tips are coloured by host from which the sample was isolated: chicken (yellow), cattle (red), pig (pink), wild bird (green), and human (black). Branches are coloured according to the ancestral source population inferred using the maximum posterior probability. Pie charts show the posterior probability for the root of the tree. For each human case, the posterior probability of source is shown as a stacked bar plot. Scale is given in units of coalescent time. Note that a change in host may have occurred at any point on the branch, not necessarily at the node, and it is also possible to have a number of host switches occurring along a branch.

Within the ST-21, ST-45 and ST-828 complexes, isolates from different host species were often more closely related than those isolated from the same host species (Figure 1). If the strains harboured previously undetected sub-ST lineages that were strongly host associated, one would expect to see distinct clusters of the same coloured branches together. However, this was not the case, with branches of the same colour—representing the reconstructed reservoir population of that lineage—scattered throughout the tree in all STs. Furthermore, one could expect that isolates from mammalian hosts would be more closely related than those from avian species, reflecting enhanced transfer potential between physiologically similar hosts. Again, this was not observed, with bird isolates in the ST-45 complex and pig isolates in ST-828 complex being closely related to both chicken and cattle isolates. This may suggest that the ability to colonize a specific host has either evolved several times throughout the tree, most plausibly through horizontal gene transfer given that mutation is rare, or that the isolates are adapted to infect all species of host in the sample.

Much of the ancestral history of the lineages was inferred to have occurred within the chicken host population, shown by the dominance of yellow branches. The most recent common ancestor of all three clonal complexes was inferred to have colonized chicken, with posterior probabilities of 0.612, 0.498 and 0.500 for the ST-21, ST-45 and ST-828 complexes, respectively.

### Rates of zoonotic transmission in *Campylobacter*

Under the host-restricted hypothesis, isolates sampled from the same source reservoir will cluster into a single clade within the phylogeny. As we sampled ST-21 complex isolates from two host species (chicken and cattle), there must have been exactly one zoonotic transfer in the tree under the restricted host hypothesis or more under the generalist model. We sampled ST-45 and ST-828 complex isolates from three host species (chicken, cattle and wild birds in ST-45 or swine in ST-828), necessitating exactly two zoonotic transfer events in the tree under the restricted host hypothesis. In fact, the total number of migration events for all three clonal complexes was estimated to be much higher than these minimum values, with 588.9 (95% credibility interval: 109.8, 1325.9), 468.7 (105.7, 1264.5) and 117.7 (36.6, 456.3) cross-species transmission events for ST-21, ST-45 and ST-828 complexes, respectively. In all three cases, the number of zoonotic transfers under the restricted host hypothesis was outside the range of the 95% credible intervals, demonstrating that isolates from these clonal complexes display host generalism and excluding the possibility of host-restricted sub-lineages.

The overall estimated migration rate was much more frequent in the *C. jejuni* clonal complexes compared with the *C. coli* (ST-828) complex. Using a mutation rate of 3.23 × 10^−5^ substitutions per site per year for calibration (Wilson *et al.*, 2009), this corresponds to approximately one host jump every 12 years for ST-828 compared with one every 1.6 or 1.8 years in the ST-21 and ST-45 complexes (Table 2). Although the credible intervals are wide, particularly for the *C. jejuni* STs, the finding of extraordinarily rapid rates of zoonotic transfer were robust to alternative prior beliefs. Thus, based upon the isolate collection in this study, the estimated rate of zoonosis is sufficiently rapid within ST-21 and ST-45 clonal complexes that there is no association between genetic structure and host species. This is consistent with a generalist lifestyle in which the isolates are equally adapted to transmission between versus within species.

### Tracing the source of human infection

**Figure 2:**
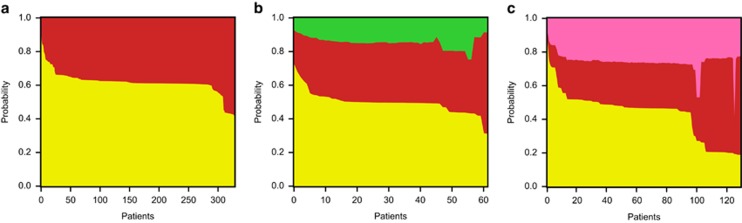
Probability of source for clinical cases for (**a**) ST-21, (**b**) ST-45 and (**c**) ST-828. The posterior probability of each human isolate (vertical bars) broken down by source population: chicken (yellow), cattle (red), pig (pink), and wild bird (green). The isolates have been reordered along the *x* axis for visualization purposes.

In Figure 1, the human cases at the tips of the tree are represented with black circles, and the posterior probability of the sources for the human isolates are illustrated by the bar plots. The majority of clinical cases (462 out of 519 cases, or 89%) were attributed to a chicken source, with 53 cases (10%) attributed to cattle and 4 (1%) to pig (Figure 2). This is consistent with results found by MLST (Wilson *et al.*, 2008; Mullner *et al.*, 2009; Sheppard *et al.*, 2009), but the difficulty of attributing individual human cases to specific source species with high confidence, even using whole genomes, reflects the remarkable transmission rates of lineages among host species. Even when human isolates were most closely related to clades sampled only from chicken, there remained appreciable probabilities (in the range 30–40%) that the direct source of transmission was non-chicken. This degree of uncertainty is comparable to the 67% accuracy of source attribution that Wilson *et al.* (2008) achieve with MLST alone.

## Discussion

We investigated the source of *Campylobacter* infection in humans using whole-genome sequencing, focussing on STs that are frequently isolated from multiple host species and are therefore weakly host associated on the basis of MLST (Wilson *et al.*, 2008; Sheppard *et al.*, 2009, 2014; Gripp *et al.*, 2011). We tested whether these STs were aggregations of strongly host-restricted sub-lineages, or whether they represented genuine generalists (Gripp *et al.*, 2011). We estimated rates of zoonotic transfer between *Campylobacter* reservoir species and attributed individual clinical cases to animal sources. Consistent with previous studies (Harris *et al.*, 1986; Wingstrand *et al.*, 2006; Wilson *et al.*, 2008; Mullner *et al.*, 2009; Sheppard *et al.*, 2009), we identified the chicken reservoir as accounting for the majority of human *Campylobacter* infections, emphasizing the importance of measures aimed at controlling food-borne disease in agriculture and the food industry.

We found that fine-scale population structure across the genome within the ST-21, ST-45 and ST-828 clonal complex isolates was not host associated. This is consistent with the existence of genuine generalist strains, adapted to transmit between and live within multiple host species. There are clear advantages of a generalist lifestyle in agricultural animal species where multiple mammalian and avian species routinely live in close proximity, giving rise to frequent opportunity for zoonotic transmission.

Some differences were observed in the relative rate of host switching between *C. coli* and *C. jejuni*. Specifically, for the *C. coli* ST-828 complex isolates, there was evidence for slower rates of zoonotic transfer, compared with the *C. jejuni* ST-21 and ST-45 complex isolates. We estimated that there were 588 migration events across the tree in the ST-21 complex and 468 in the ST-45 complex, compared with only 117 in ST-828. In all cases, we were able to reject the hypothesis of a unique host jump founding the population in each new species, with significantly more migration events than would be expected if isolates were host restricted (Table 2). This is evidenced by the scattering of isolates sampled from different sources throughout the phylogeny.

The overall rates of migration between different host species provides information about the ecology of the generalist *Campylobacter* strains. Within the ST-45 complex, there was very little difference in the relative rates of transmission between host species (Table 2). However, in ST-828 complex isolates, host switches were twice as frequent between cattle and swine compared with the rates of migration between these species and chicken. This disparity between mammal–mammal and mammal–bird transmission rates suggests that the efficacy of zoonotic transmission may be lower in the ST-828 complex compared with ST-21 or ST-45 complexes. Mammals and birds exhibit many physiological differences, including a difference in core body temperature of about 38 °C in cattle and pigs versus 42 °C in chickens. Variation in rates of zoonosis may be explained by differences in the route of transmission between the mammalian species, either through opportunity—for example, if cattle and pigs have more commonly shared the same physical environment—or through affinity—involving factors associated with the ability to colonize different species. Suggestively, the average genome size of ST-828 complex isolates is smaller than that of ST-21 and ST-45 complex isolates, potentially reflecting more limited phenotypic plasticity that would limit the ability to occupy multiple divergent niches.

The environment is not thought to be important as a reservoir for clinical disease, and thus we did not explicitly include it in the model here. Current research suggests that when *Campylobacter* is sampled outside of the host it is typically associated with faecal contamination, with no active proliferation outside the gut. Such environmental isolates would be found associated with their most recent host. However, this does not exclude the environment as an intermediate link for zoonosis; rather, the host switching rate between two species includes the whole process of zoonosis from one to the other.

The use of a mutation rate, calibrated in calendar time units, allowed us to calibrate migration rates into calendar time. This was not possible to estimate directly from the data, owing to the lack of longitudinal sample information. The information on mutation rates in *Campylobacter* is limited, leading to us using a rate estimated from *C. jejuni* MLST data. Whole-genome sequencing has demonstrated that mutation rates in bacteria are typically on the order of 10^−7^−10^−5^ per site per year (Didelot *et al.*, 2012), which is consistent with rates estimated by MLST (Falush *et al.*, 2001; Pérez-Losada *et al.*, 2007; Wilson *et al.*, 2009). Because of the generalization of this point estimate to the rest of the coding genome and *C. coli*, we note that there are limitations to the estimated host switching rates and time to most recent common ancestor. The branch lengths of the tree may also have been skewed by the removal of homoplasious sites (Hedge and Wilson, 2014). However, the branch lengths are of limited direct interest, and we do not expect them to strongly influence estimation of relative rates of transmission between host species as the topology continues to show high accuracy to the clonal frame.

By comparing an approach using relatively few isolates characterized at many loci, by whole-genome sequencing, with methods using hundreds or thousands of isolates characterized at few loci, such as MLST studies (Wilson *et al.*, 2008; Mullner *et al.*, 2009; Sheppard *et al.*, 2009), we investigated the added benefit of whole-genome sequencing for investigating lineages with rapid host switching within clonal complexes. In the majority of human cases, there was little additional information regarding source of infection provided by analysing the entire coding element of the *Campylobacter* genome, identified in the reference strain (Parkhill *et al.*, 2000; Gundogdu *et al.*, 2007). This suggests an inherent trade-off between the number of samples and the number of loci sequenced and that the approximately thousand-fold increase in sequence information afforded by whole genomes over seven-locus MLST does not, on its own, eliminate the need for detailed sampling. While there is evidence of the potential for whole-genome sequencing to provide greater resolution in detecting recent transmission of human *Campylobacter* infection from non-chicken sources, and accessory genome elements may provide host-specific information (Sheppard *et al.*, 2013b), this study highlights the importance of intensive sampling of potential source populations and an understanding of host transmission ecology for effective source attribution.

## Figures and Tables

**Table 1:** Summary of sequence data for the three clonal complexes

	*ST-21 complex*	*ST-45 complex*	*ST-828 complex*
*Genome alignment length*	1 544 595	1 465 323	1 421 603
Non-polymorphic sites	1 447 536	1 386 349	1 272 113
Polymorphic sites	97 059	78 929	149 490
*Biallelic sites*	90 239	722 233	136 375
Compatible with ML tree	41 895	34 960	60 692
			
Total sites used in analysis	1 489 431	1 421 309	1 332 805

Abbreviation: ML, maximum likelihood.

**Table 2:** Parameter estimates for each clonal complex

*Parameter*	*ST-21 complex*	*ST-45 complex*	*ST-828 complex*
Substitution rate (10^−3^ per site per τ)	2.815 (1.950, 3.560)	3.715 (2.315, 5.158)	9.582 (8.019, 11.534)
			
*TMRCA*			
Coalescent time, τ	1.022 (0.442, 3.068)	0.709 (0.288, 2.583)	0.159 (0.110, 0.232)
Years^a^	89.069 (38.521, 267.381)	81.545 (33.124, 297.085)	47.168 (32.632, 68.824)
			
*Host switching rate*			
Per τ	54.078 (10.089,97.519)	61.634 (14.540, 98.069)	23.491 (6.802, 85.357)
Per year^a^	0.621 (0.116, 1.119)	0.536 (0.126, 0.853)	0.079 (0.023, 0.287)
			
Number of migrations	588.89 (109.80, 1325.90)	468.67 (105.70, 1264.48)	117.691 (36.558, 456.318)
			
*Estimated host frequencies*			
Chicken	0.614 (0.388, 0.809)	0.498 (0.311, 0.685)	0.425 (0.136, 0.691)
Cattle	0.386 (0.191, 0.612)	0.348 (0.185, 0.540)	0.282 (0.095, 0.562)
Wild bird	—	0.140 (0.0429, 0.313)	—
Pig	—	—	0.270 (0.103, 0.545)
			
*Relative migration rates between host species*			
Chicken–cattle	0.695 (0.023, 3.705)	0.820 (0.051, 3.779)	0.303 (0.010, 2.139)
Chicken–wild bird	—	0.592 (0.021, 3.282)	—
Chicken–pig	—	—	0.581 (0.024, 3.129)
Cattle–wild bird	—	0.728 (0.031, 3.643)	—
Cattle–pig	—	—	1.367 (0.192, 4.783)

Abbreviation: TMRCA, time to most recent common ancestor.

a Using the mutation rate 3.23 × 10^−5^ substitutions per site per year for calibration with the median evolutionary rate from the BEAST analysis, one unit of coalescent time (τ) is equal to: 87.151 years in the ST-21 complex, 115.015 years in ST-45 complex and 296.656 years in ST-828 complex. The median value was used for estimating all other parameters in years.

## References

[bib1] Allos BM. (2001). *Campylobacter jejuni* infections: update on emerging issues and trends. Clin Infect Dis 32: 1201–1206. 11283810 10.1086/319760

[bib2] Clark CG, Price L, Ahmed R, Woodward DL, Melito PL, Rodgers FG et al. (2003). Characterization of waterborne outbreak-associated *Campylobacter jejuni*, Walkerton, Ontario. Emerg Infect Dis 9: 1232–1241. 14609457 10.3201/eid0910.020584 PMC3033067

[bib3] Cody AJ, McCarthy ND, Jansen van Rensburg M, Isinkaye T, Bentley SD, Parkhill J et al. (2013). Real-time genomic epidemiological evaluation of human *Campylobacter* isolates by use of whole-genome multilocus sequence typing. J Clin Microbiol 51: 2526–2534. 23698529 10.1128/JCM.00066-13 PMC3719633

[bib4] Didelot X, Bowden R, Wilson DJ, Peto TEA, Crook DW. (2012). Transforming clinical microbiology with bacterial genome sequencing. Nat Rev Genet 13: 601–612. 22868263 10.1038/nrg3226 PMC5049685

[bib5] Didelot X, Falush D. (2007). Inference of bacterial microevolution using multilocus sequence data. Genetics 175: 1251–1266. 17151252 10.1534/genetics.106.063305 PMC1840087

[bib6] Didelot X, Wilson DJ. (2015). ClonalFrameML: efficient inference of recombination in whole bacterial genomes. PLOS Comput Biol 11: e1004041. 25675341 10.1371/journal.pcbi.1004041 PMC4326465

[bib7] Drummond AJ, Ho SYW, Phillips MJ, Rambaut A. (2006). Relaxed phylogenetics and dating with confidence. PLoS Biol 4: e88. 16683862 10.1371/journal.pbio.0040088 PMC1395354

[bib8] Drummond AJ, Suchard MA, Xie D, Rambaut A. (2012). Bayesian phylogenetics with BEAUti and the BEAST 1.7. Mol Biol Evol 29: 1969–1973. 22367748 10.1093/molbev/mss075 PMC3408070

[bib9] Edgar RC. (2004). MUSCLE: multiple sequence alignment with high accuracy and high throughput. Nucleic Acids Res 32: 1792–1797. 15034147 10.1093/nar/gkh340 PMC390337

[bib10] Engberg J, Gerner-Smidt P, Scheutz F, Møller Nielson E, On SLW, Mølbak K. (1998). Water-borne *Campylobacter jejuni* infection in a Danish town - a 6-week continuous source outbreak. Clin Microbiol Infect 4: 648–656. 11864264 10.1111/j.1469-0691.1998.tb00348.x

[bib11] Eyre DW, Cule ML, Wilson DJ, Griffiths D, Vaughan A, O'Connor L et al. (2013). Diverse sources of *C. difficile* infection identified on whole-genome sequencing. N Engl J Med 369: 1195–1205. 24066741 10.1056/NEJMoa1216064 PMC3868928

[bib12] Falush D, Kraft C, Taylor NS, Correa P, Fox JG, Achtman M et al. (2001). Recombination and mutation during long-term gastric colonization by Helicobacter pylori: estimates of clock rates, recombination size, and minimal age. Proc Natl Acad Sci USA 98: 15056–15061. 11742075 10.1073/pnas.251396098 PMC64982

[bib13] Griekspoor P, Colles FM, McCarthy ND, Hansbro PM, Ashhurst-Smith C, Olsen B et al. (2013). Marked host specificity and lack of phylogeographic population structure of *Campylobacter jejuni* in wild birds. Mol Ecol 22: 1463–1472. 23356487 10.1111/mec.12144 PMC3596980

[bib14] Gripp E, Hlahla D, Didelot X, Kops F, Maurischat S, Tedin K et al. (2011). Closely related *Campylobacter jejuni* strains from different sources reveal a generalist rather than a specialist lifestyle. BMC Genomics 12: 584. 22122991 10.1186/1471-2164-12-584 PMC3283744

[bib15] Guindon S, Dufayard J-F, Lefort V, Anisimova M, Hordijk W, Gascuel O. (2010). New algorithms and methods to estimate maximum-likelihood phylogenies: assessing the performance of PhyML 3.0. Syst Biol 59: 307–321. 20525638 10.1093/sysbio/syq010

[bib16] Gundogdu O, Bentley SD, Holden MT, Parkhill J, Dorrell N, Wren BW. (2007). Re-annotation and re-analysis of the *Campylobacter jejuni* NCTC11168 genome seq.uence. BMC Genomics 8: 162. 17565669 10.1186/1471-2164-8-162 PMC1899501

[bib17] Harris N V, Weiss NS, Nolan CM. (1986). The role of poultry and meats in the etiology of *Campylobacter jejuni/coli* enteritis. Am J Public Health 76: 407–411. 3953917 10.2105/ajph.76.4.407 PMC1646506

[bib18] Harris SR, Feil EJ, Holden MTG, Quail MA, Nickerson EK, Chantratita N et al. (2010). Evolution of MRSA during hospital transmission and intercontinental spread. Science 327: 469–474. 20093474 10.1126/science.1182395 PMC2821690

[bib19] Hasegawa M, Kishino H, Yano T. (1985). Dating of the human-ape splitting by a molecular clock of mitochondrial DNA. J Mol Evol 22: 160–174. 3934395 10.1007/BF02101694

[bib20] Hedge J, Wilson DJ. (2014). Bacterial phylogenetic reconstruction from whole genomes is robust to recombination but demographic inference is not. MBio 5: e02158. 25425237 10.1128/mBio.02158-14 PMC4251999

[bib21] Jolley KA, Maiden MCJ. (2010). BIGSdb: scalable analysis of bacterial genome variation at the population level. BMC Bioinformatics 11: 595. 21143983 10.1186/1471-2105-11-595 PMC3004885

[bib22] Lefébure T, Pavinski Bitar PD, Suzuki H, Stanhope MJ. (2010). Evolutionary dynamics of complete *Campylobacter* pan-genomes and the bacterial species concept. Genome Biol Evol 2: 646–655. 20688752 10.1093/gbe/evq048 PMC2940326

[bib23] Lemey P, Rambaut A, Drummond AJ, Suchard MA. (2009). Bayesian phylogeography finds its roots. PLoS Comput Biol 5: e1000520. 19779555 10.1371/journal.pcbi.1000520 PMC2740835

[bib24] McCarthy ND, Colles FM, Dingle KE, Bagnall MC, Manning G, Maiden MCJ et al. (2007). Host-associated genetic import in *Campylobacter jejuni*. Emerg Infect Dis 13: 267–272. 17479890 10.3201/eid1302.060620 PMC2063414

[bib25] Minin VN, Suchard MA. (2008a). Counting labeled transitions in continuous-time Markov models of evolution. J Math Biol 56: 391–412. 17874105 10.1007/s00285-007-0120-8

[bib26] Minin VN, Suchard MA. (2008b). Fast, accurate and simulation-free stochastic mapping. Phil Trans R Soc B 363: 3985–3995. 18852111 10.1098/rstb.2008.0176 PMC2607419

[bib27] Mullner P, Spencer SEF, Wilson DJ, Jones G, Noble AD, Midwinter AC et al. (2009). Assigning the source of human campylobacteriosis in New Zealand: a comparative genetic and epidemiological approach. Infect Genet Evol 9: 1311–1319. 19778636 10.1016/j.meegid.2009.09.003

[bib28] Neimann J, Engberg J, Mølbak K, Wegener HC. (2003). A case-control study of risk factors for sporadic campylobacter infections in Denmark. Epidemiol Infect 130: 353–366. 12825719 10.1017/s0950268803008355 PMC2869971

[bib29] Palmer SR, Gully PR, White JM, Person AD, Suckling WG, Jones DM et al. (1983). Water-borne outbreak of *Campylobacter* gastroenteritis. Lancet 321: 287–290. 10.1016/s0140-6736(83)91698-7 6130305

[bib30] Parkhill J, Wren BW, Mungall K, Ketley JM, Churcher C, Basham D et al. (2000). The genome sequence of the food-borne pathogen *Campylobacter jejuni* reveals hypervariable sequences. Nature 403: 665–668. 10688204 10.1038/35001088

[bib31] Pebody RG, Ryan MJ, Wall PG. (1997). Outbreaks of campylobacter infection: rare events for a common pathogen. Commun Dis Rep Rev 7: R33–R37. 9080726

[bib32] Pérez-Losada M, Crandall KA, Zenilman J, Viscidi RP. (2007). Temporal trends in gonococcal population genetics in a high prevalence urban community. Infect Genet Evol 7: 271–278. 17141576 10.1016/j.meegid.2006.11.003 PMC1820634

[bib33] Price JR, Golubchik T, Cole K, Wilson DJ, Crook DW, Thwaites GE et al. (2014). Whole-genome sequencing shows that patient-to-patient transmission rarely accounts for acquisition of *Staphylococcus aureus* in an intensive care unit. Clin Infect Dis 58: 609–618. 24336829 10.1093/cid/cit807 PMC3922217

[bib34] Pupko T, Pe'er I, Shamir R, Graur D. (2000). A fast algorithm for joint reconstruction of ancestral amino acid sequences. Mol Biol Evol 17: 890–896. 10833195 10.1093/oxfordjournals.molbev.a026369

[bib35] Sears A, Baker MG, Wilson N, Marshall J, Muellner P, Campbell DM et al. (2011). Marked Campylobacteriosis decline after interventions aimed at poultry, New Zealand. Emerg Infect Dis 17: 1007–1015. 21749761 10.3201/eid1706.101272 PMC3358198

[bib36] Sheppard SK, Cheng L, Méric G, de Haan CPA, Llarena A-K, Marttinen P et al. (2014). Cryptic ecology among host generalist *Campylobacter jejuni* in domestic animals. Mol Ecol 23: 2442–2451. 24689900 10.1111/mec.12742 PMC4237157

[bib37] Sheppard SK, Colles FM, McCarthy ND, Strachan NJC, Ogden ID, Forbes KJ et al. (2011). Niche segregation and genetic structure of *Campylobacter jejuni* populations from wild and agricultural host species. Mol Ecol 20: 3484–3490. 21762392 10.1111/j.1365-294X.2011.05179.x PMC3985062

[bib38] Sheppard SK, Dallas JF, Strachan NJC, MacRae M, McCarthy ND, Wilson DJ et al. (2009). *Campylobacter* genotyping to determine the source of human infection. Clin Infect Dis 48: 1072–1078. 19275496 10.1086/597402 PMC3988352

[bib39] Sheppard SK, Didelot X, Jolley KA, Darling AE, Pascoe B, Meric G et al. (2013a). Progressive genome-wide introgression in agricultural *Campylobacter coli*. Mol Ecol 22: 1051–1064. 23279096 10.1111/mec.12162 PMC3749442

[bib40] Sheppard SK, Didelot X, Meric G, Torralbo A, Jolley KA, Kelly DJ et al. (2013b). Genome-wide association study identifies vitamin B5 biosynthesis as a host specificity factor in *Campylobacter*. Proc Natl Acad Sci USA 110: 11923–11927. 23818615 10.1073/pnas.1305559110 PMC3718156

[bib41] Sheppard SK, Maiden MCJ, Falush D (2010). Population genetics of Campylobacter. In: Robinson DA, Falush D, Feil EJ (eds). Bacterial Populations in Infectious Disease. Wiley-Blackwell: Hoboken, NJ, USA, pp 184.

[bib42] Talbi C, Lemey P, Suchard MA, Abdelatif E, Elharrak M, Nourlil J et al. (2010). Phylodynamics and human-mediated dispersal of a zoonotic virus. PLoS Pathog 6: e1001166. 21060816 10.1371/journal.ppat.1001166 PMC2965766

[bib43] Walker TM, Ip CLC, Harrell RH, Evans JT, Kapatai G, Dedicoat MJ et al. (2013). Whole-genome sequencing to delineate *Mycobacterium tuberculosis* outbreaks: a retrospective observational study. Lancet Infect Dis 13: 137–146. 23158499 10.1016/S1473-3099(12)70277-3 PMC3556524

[bib44] Wilson DJ, Gabriel E, Leatherbarrow AJH, Cheesbrough J, Gee S, Bolton E et al. (2009). Rapid evolution and the importance of recombination to the gastroenteric pathogen *Campylobacter jejuni*. Mol Biol Evol 26: 385–397. 19008526 10.1093/molbev/msn264 PMC2639114

[bib45] Wilson DJ, Gabriel E, Leatherbarrow AJH, Cheesbrough J, Gee S, Bolton E et al. (2008). Tracing the source of campylobacteriosis. PLoS Genet 4: e1000203. 18818764 10.1371/journal.pgen.1000203 PMC2538567

[bib46] Wingstrand A, Neimann J, Engberg J, Møller Nielsen E, Gerner-Smidt P, Wegener HC et al. (2006). Fresh chicken as main risk factor for campylobacteriosis, Denmark. Emerg Infect Dis 12: 280–284. 16494755 10.3201/eid1202.050936 PMC3373097

[bib47] Zerbino DR, Birney E. (2008). Velvet: algorithms for de novo short read assembly using de Bruijn graphs. Genome Res 18: 821–829. 18349386 10.1101/gr.074492.107 PMC2336801

