# Supplementary material for: Rapid host switching in generalist Campylobacter strains erodes the signal for tracing human infections
Source: ISME J. 2015 Aug 25;10(3):721–9. doi: 10.1038/ismej.2015.149 (PMC4677457; doi:10.1038/ismej.2015.149)
Supplement: Supplementary Information [file ismej2015149x1.doc]

**Supplementary Table 1: Isolates included in the study.**

All isolates are available from <http://pubmlst.org/campylobacter/>.

*Sequence type was derived from the allelic profile of seven housekeeping genes by multilocus sequence typing (MLST).

**Clonal complex is defined as including any ST that matches a previously defined central genotype (<http://pubmlst.org/campylobacter/>) at three or more loci.

***References refer to:

1. Sheppard SK, Didelot X, Jolley KA, Darling AE, Pascoe B, Meric G, Kelly DJ, Cody A, Colles FM, Strachan NJC, Ogden ID, Forbes K, French NP, Carter P, Miller WG, McCarthy ND, Owen R, Litrup E, Egholm M, Affourtit JP, Bentley SD, Parkhill J, Maiden MCJ, Falush D (2013) Progressive genome-wide introgression in agricultural *Campylobacter coli*. *Mol Ecol*, **22**:1051–64.

2. Sheppard SK, Didelot X, Meric G, Torralbo A, Jolley KA, Kelly DJ, Bentley SD, Maiden MCJ, Parkhill J, Falush D (2013) Genome-wide association study identifies vitamin B5 biosynthesis as a host specificity factor in Campylobacter. *Proc Natl Acad Sci USA*, **110**:11923–11927.

3. Sheppard SK, Cheng L, Méric G, de Haan CPA, Llarena A-K, Marttinen P, Vidal A, Ridley A, Clifton-Hadley F, Connor TR, Strachan NJC, Forbes K, Colles FM, Jolley KA, Bentley SD, Maiden MCJ, Hänninen M-L, Parkhill J, Hanage WP, Corander J (2014) Cryptic ecology among host generalist *Campylobacter jejuni* in domestic animals. *Mol Ecol*, **23**:2442–51.

4. Lefébure T, Pavinski Bitar PD, Suzuki H, Stanhope MJ (2010) Evolutionary dynamics of complete *Campylobacter* pan-genomes and the bacterial species concept. *Genome Biol Evol*, **2**:646–55.

5. Cody AJ, McCarthy ND, Jansen van Rensburg M, Isinkaye T, Bentley SD, Parkhill J, Dingle KE, Bowler ICJW, Jolley KA, Maiden MCJ (2013) Real-time genomic epidemiological evaluation of human Campylobacter isolates by use of whole-genome multilocus sequence typing. *J Clin Microbiol*, **51**:2526–34.
